# Resolutions

**Published:** 1806-06

**Authors:** 


### RESOLUTIONS.

We, the undersigned, being impressed with a strong conviction, that the advantages of Vaccine Inoculation are, in many important circumstances, greatly superior to those of inoculation for the Small-pox: and being desirous of extending and diffusing these advantages as widely as possible in this town and neighbourhood, where Vaccination has hitherto been practised to a less extent, in proportion to the population, than in many other parts of this kingdom, and even of this county, have agreed to the following Resolutions, as declaratory of our sentiments on this most interesting subject, viz.

Resolution 1st. That, in our opinion, inoculation for the Cow-pock

Cow-pock affords to the human constitution a protection against the contagion of the Small-pox, as complete and effectual as can possibly be derived from inoculation for the Small-pox.

Resolution 2d. That, in our opinion, inoculation for the Cow-pock is greatly preferable to inoculation for the Small-pox, for the following reasons:

1st. Because the disease produced by Vaccination not only is never fatal, but is never even attended with danger; whilst, on the contrary, it is well known, that, of such as have been inoculated for the Small-pox, some have occasionally died, notwithstanding every precaution; and it is equally notorious, that in those who have passed through the inoculated Small-pox, the disease has been sometimes dangerous and severe, and its effects on the constitution have been often permanently injurious, by exciting other diseases into action.

2dly, Because the Vaccine disease is not infectious, as the Small-pox always is, whether it be received naturally, or by inoculation.

3dly, Because it may be communicated with perfect safety, under circumstances which render Small-pox inoculation peculiarly formidable; as, in the state of pregnancy, and during infancy, and the period of dentition.

And lastly, Because it has never been followed, in any instance that has yet come to our knowledge, by any effects of a serious nature. Cutaneous eruptions have occurred in a few instances after Vaccination here as well as elsewhere; but it has by no means been proved, that such eruptions are fairly to be ascribed to Vaccination as their cause. And in this opinion we are confirmed by the public testimony of the ablest and most respectable practitioners in the metropolis, who have paid close attention to this subject, and who have declared, that "many well known cutaneous diseases, and some scrophulous complaints, have been represented as the effects of Vaccine Inoculation, when, in fact, they have originated from other causes, and, in many instances, have occurred long after vaccination; and that such diseases are infinitely less frequent after Vaccination, than after either the natural or inoculated Small-pox."

Resolution 3d. That, influenced by these powerful considerations, we feel it to be our incumbent duty to discourage the practice of inoculation for the Small-pox, being firmly convinced, that it does not present to us one single advantage which cannot be obtained with equal certainty, and without any danger, by Vaccine Inoculation; and that it prolongs the existence, and extends the ravages, of a most destructive disease, which Vaccine Inoculation promises ultimately to exterminate.

As an additional reason for this Resolution, we think it necessary to state, that it appears from authentic documents, and from calculations founded upon them, which were produced in evidence by respectable professional Gentlemen, when they were examined on this subject by a Committee of the House of Commons, in the year 1802, that the mortality of the small-pox has been increased since the introduction of inoculation. And this mortality has prevailed to such an extent, that not less than 34,000 individuals have annually perished by that disease alone, in the United Kingdom of Great Britain and Ireland.

For these reasons we give a decided preference to Vaccine Inoculation; and we are determined to use all our influence to promote the practice of it in this town and neighbourhood as extensively as possible, amongst all ranks and classes of society.

John Ball
Edward Barnes
Francis Baron
Oliver Barron, M. D.
W. Barrow, M. D.
S. Beetenson, M. D.
John Bostock, M. D.
Joseph Brandreth, M. D.
Jos. P. Brandreth, M. B.
Joseph Brandreth
W. Briggs, M. D.
S. Bromley
Rt. Buddicom
Robt. Buchanan, M. D.
Christ. Buck
Jas. Carson, M. D.
Thomas Christian
Law. Cotham
John P. Dale
James Dawson
Robt. Fleetwood
Richard Forshaw
James Gerard, M. D.
W. Gresley
Thos. Fairfax Hay
H. B. Hensman
Thomas Houghton
John Hicks
L. J. Jardine, M. D.
Thos. Jeffries, M. D.
Robert Lewin, M. D.
James C. Lynch, M. D.
Peter Lindsay
John Lyon, M. D.
John M'Cartney, M. D.
George Mather
John M'Cullock
Patrick M'Dermot
John M'Gowan
Henry Park
Wm. Perry
J. W. Pursell, M. D.
Wm. Lucas Reay
Thomas Renwick, M. D.
John Rutter, M. D.
John Shaw
C. Shuttleworth
Robert Simon
O. Thomas
M. Timons
Tho. Stewart Traill, M. D.
C. Worthington
Jos. Vigneaux

